# Self-oscillating chemoelectrical interface of solution-gated ion-sensitive field-effect transistor based on Belousov–Zhabotinsky reaction

**DOI:** 10.1038/s41598-022-06964-4

**Published:** 2022-02-22

**Authors:** Toshiya Sakata, Shoichi Nishitani, Yusuke Yasuoka, Shogo Himori, Kenta Homma, Tsukuru Masuda, Aya Mizutani Akimoto, Kazuaki Sawada, Ryo Yoshida

**Affiliations:** 1 Department of Materials Engineering, School of Engineering, The University of Tokyo, 7-3-1 Hongo, Bunkyo-ku, Tokyo, 113-8656 Japan; 2 Department of Bioengineering, School of Engineering, The University of Tokyo, 7-3-1 Hongo, Bunkyo-ku, Tokyo, 113-8656 Japan; 3 Department of Electrical and Electronic Information Engineering, Toyohashi University of Technology, 1-1 Hibarigaoka, Tempaku-cho, Toyohashi, Aichi, 441-8580 Japan

**Keywords:** Materials science, Materials for devices, Techniques and instrumentation, Analytical chemistry, Electrochemistry

## Abstract

The Belousov–Zhabotinsky (BZ) self-oscillation reaction is an important chemical model to elucidate nonequilibrium chemistry in an open system. However, there are only a few studies on the electrical behavior of pH oscillation induced by the BZ reaction, although numerous studies have been carried out to investigate the mechanisms by which the BZ reaction interacts with redox reactions, which results in potential changes. Needless to say, the electrical characteristic of a self-oscillating polymer gel driven by the BZ reaction has not been clarified. On the other hand, a solution-gated ion-sensitive field-effect transistor (ISFET) has a superior ability to detect ionic charges and includes capacitive membranes on the gate electrode. In this study, we carried out the electrical monitoring of self-oscillation behaviors at the chemoelectrical interface based on the BZ reaction using ISFET sensors, focusing on the pH oscillation and the electrical dynamics of the self-oscillating polymer brush. The pH oscillation induced by the BZ reaction is not only electrically observed using the ISFET sensor, the electrical signals of which results from the interfacial potential between the solution and the gate insulator, but also visualized using a large-scale and high-density ISFET sensor. Moreover, the *N*-isopropylacrylamide (NIPAAm)-based self-oscillating polymer brush with Ru(bpy)_3_ as a catalyst clearly shows a periodic electrical response based on the swelling–deswelling behavior caused by the BZ reaction on the gate insulator of the ISFET sensor. Thus, the elucidation of the electrical self-oscillation behaviors induced by the BZ reaction using the ISFET sensor provides a solution to the problems of nonequilibrium chemistry.

## Introduction

As a chemical model of the tricarboxylic acid cycle (TCA), the Belousov–Zhabotinsky (BZ) reaction is well known to show a chemical self-oscillation behavior that is not dominated by equilibrium thermodynamic behavior (nonequilibrium chemistry)^1–3^. In general, a reactant changes to a reaction product in one direction or in an equilibrium state through a chemical reaction. However, a self-oscillation reaction can be chemically induced under an optimum condition, where the reaction products (intermediates) are periodically utilized as the reactants. In the BZ reaction, organic substances such as malonic acid reduces metallic catalysts (e.g., Ce^4+^, Fe(phen)_3_^3+^, Ru(bpy)_3_^3+^), and then their reduced catalysts are oxidized by oxidants such as bromic acid (BrO_3_^-^) in an acid solution, where the cyclic redox reaction spontaneously occurs. When the ruthenium bipyridine complex Ru(bpy)_3_^2+/3+^ is utilized as a metallic catalyst, the cyclic redox reaction is observed from the change in the color of the BZ solution [Ru(bpy)_3_^2+^ (orange) $\rightleftarrows$ Ru(bpy)_3_^3+^ (light green) + e^−^]^4,5^. This cyclic redox reaction was also electrochemically monitored using metal electrodes such as Pt and Ag on the basis of the half-cell reaction^6^. Simultaneously, the concentration of protons, [H^+^], should periodically change in accordance with the BZ reaction. In fact, there are a few studies in which the change in pH caused by the BZ reaction was investigated using ion sensitive electrodes^7,8^. On the other hand, the changes in the concentrations of catalysts and intermediates such as Br^−^ seemed to have been analyzed without considering the change in [H^+^] (pH) in many studies^9–14^. However, the cyclic behavior of pH based on the BZ reaction was not necessarily observed using an ion sensitive array^7^, nor was it imaged using an ion-sensitive field-effect transistor (ISFET)^8^, which is utilized in this study. Therefore, the monitoring and imaging of the pH self-oscillation based on the BZ reaction is valuable for the elucidation of nonequilibrium reactions.

The BZ reaction proceeds in a nonlinear chemical reaction system under open and batch conditions^15,16^. A polymer gel is a cross-linked three-dimensional polymer network that swells upon immersion in a solvent, providing an open system for heat and mass transfer. So far, we have found a self-oscillating system in a stimulus-responsive polymer gel based on *N*-isopropylacrylamide (NIPAAm) that is driven by the BZ reaction. In particular, the poly[NIPAAm-*co*-Ru(bpy)_3_] gel clearly exhibited periodical volume changes (swelling and deswelling) in an aqueous solution containing the reactants of the BZ reaction, except for the catalyst^5,17^. This indicates that the chemomechanical self-oscillation of the polymer gel is realized in a closed solution without external stimuli. Therefore, the self-oscillating polymer gel is expected to be applied to the observation of peristatic motions in biological systems and actuators in bioanalytical systems^18–20^. In addition, the chemomechanical energy derived from the self-oscillating polymer gel may be converted into electrical energy for autonomous sensors when its electrical properties are clarified.

Considering the above, we examined the self-oscillating chemoelectrical interface based on the BZ reaction using a solution-gated ISFET sensor. This sensor was proposed for detecting ions and biomolecules in biological environments^21,22^. In this device, an electrolyte solution induces the interfacial potential between the solution and the gate insulator instead of a metal gate in a metal–oxide–semiconductor (MOS) transistor; this device, however, requires a reference electrode in the solution. The gate insulator is often composed of oxide or nitride membranes such as Ta_2_O_5_, Al_2_O_3_, Si_3_N_4_, and SiO_2_; therefore, hydroxyl groups at the oxide or nitride surface in the solution undergo the equilibrium reaction with hydrogen ions through protonation (–OH + H^+^ $\rightleftarrows$ –OH_2_^+^) and deprotonation (–OH $\rightleftarrows$ –O^−^ + H^+^). This is why the change in the surface charge is detected from the change in pH without being mostly affected by other cations in accordance with the principle of the field effect^23,24^. Such ISFET sensors mostly have a silicon substrate, but a variety of semiconducting materials have recently been applied to pH-sensitive ISFET sensors^25,26^. Moreover, a large-scale and high-density ISFET (arrayed-gate ISFET) sensor has recently been developed with the progress of complementary MOS (CMOS) integrated circuit technologies^27–34^. Using the arrayed-gate ISFET sensor, one can image the change in pH in the solution by detecting it on each gate insulator, which is called the pH image sensor^27^. In a previous paper, an ideal pH sensitivity of approximately 55 mV/pH, which was near the Nernstian response at the electrolyte solution/gate insulator interface, and a high frame speed of 1933 fps were reported^34^. Notably, this sensor had the smallest pitch of 2 mm, which resulted in a high spatial resolution.

In addition, the gate insulator surface of the ISFET sensor was chemically modified to specifically and selectively detect biomolecules and to reduce electrical noise from interfering species^35,36^. Also, such chemical modifications on the gate insulator surface contributed to the electrical detection of the phase transition (swelling and deswelling) in the poly(NIPAAm) gel^37^. Therefore, the self-oscillating polymer gel-coated gate ISFET sensor is expected to be used for the electrical monitoring of self-oscillation based on the phase transition on the modified surface, which is driven by the BZ reaction.

In this paper, we report the electrical monitoring of self-oscillation at the chemoelectrical interface based on the BZ reaction using the ISFET sensors, focusing on the pH oscillation and the electrical dynamics of the self-oscillating polymer brush.

## Results and discussion

### Real-time monitoring of pH oscillation induced by BZ reaction with single-gate ISFET sensor

**Figure 1:**
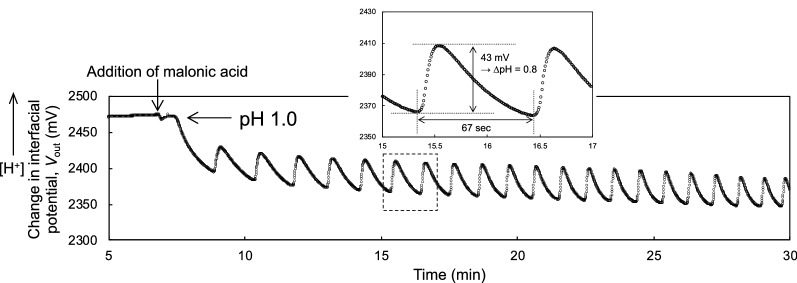
Change in interfacial potential (Δ*V*_out_) for BZ reaction detected using single-gate ISFET sensor. Malonic acid was added to the BZ presolution without malonic acid (pH 1.0). Δ*V*_out_ was translated to pH in accordance with the pH sensitivity of the ISFET sensor (i.e., the Nernstian response) used in this study (Fig. S1).

**Figure 2:**
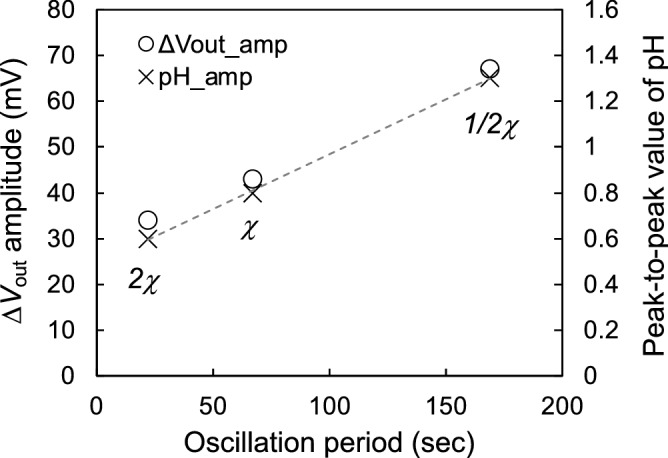
Peak-to-peak potential and pH (amplitude) for oscillation period computed on the basis of Figs. 1 and S2. The concentration χ of the BZ solution was controlled to 1/2χ, χ, and 2χ. The oscillation periods, Δ*V*_out_, and ΔpH were averaged in the limited range of about 5 min. Each standard error shows < 0.3 s for the oscillation periods, < 0.5 mV for Δ*V*_out_, and < 0.01 for ΔpH.

The Ta_2_O_5_ film was used as the gate insulator of the ISFET sensor in this study. The Ta_2_O_5_ surface has hydroxy groups in an electrolyte solution, which shows the equilibrium reaction with hydrogen ions depending on pH. Therefore, the change in potential at the solution/Ta_2_O_5_ gate insulator interface is electrically output with varying pHs, depending on the Nernstian response (59.2 mV/pH at 25 ℃). The Nernst equation for pH is shown as Eq. 1 and strictly reflected by the coefficient [*β*/(*β* + 1)] with the parameter *β* (Eq. 2):

1 $$\varphi_{0} = 2.303\frac{kT}{q}\left( {\frac{\beta }{\beta + 1}} \right)\left( {{\text{pH}}_{{{\text{pzc}}}} - {\text{pH}}} \right)$$

2 $${\text{with}}\quad \beta { } = \frac{{2q^{2} N_{{\text{S}}} K_{{\text{a}}}^{1/2} }}{{kTC_{{{\text{EDL}}}} }}$$

where *φ*_0_ is the interfacial potential at an electrolyte solution/gate insulator interface, pH_pzc_ is the point of zero charge, *q* is the elementary charge, *N*_S_ is the site density of hydroxyl groups at the oxide membrane, *K*_*a*_ is the equilibrium constant between hydrogen ions and hydroxyl groups, *k* is the Boltzmann constant, *T* is the absolute temperature, and *C*_EDL_ is the capacitance of the electric double layer^21^. The Ta_2_O_5_-gate ISFET shows higher pH sensitivity near the ideal sensitivity owing to the higher density of hydroxy groups at the Ta_2_O_5_ surface (ca. 10^15^/cm^2^)^38^, which is sufficiently high for *β*/(*β* + 1) to be assumed as 1. On the other hand, the BZ reaction is induced under a strongly acidic condition. This is why the change in pH during the BZ oscillation may have never been expected. However, the pH oscillation was induced upon adding malonic acid to the BZ presolution (malonic-acid-free) and was clearly detected using the single-gate ISFET sensor, as shown in Fig. 1. The actual pH was estimated from the calibration curve (Δ*V*_out_ with ΔpH) of the single-gate ISFET sensor used in this study (Fig. S1). The average of the pH sensitivity was $55.2 \pm 2.1$ mV/pH (n = 10) and then the pH of the BZ presolution was determined to be around 1.0 (Fig. S1). In the BZ solution with the concentration of *χ*, the peak-to-peak Δ*V*_out_ $\left( {\Delta V_{{{\text{out}}}}^{{{\text{peak}}}} } \right)$ was approximately 43 mV, which corresponded to ΔpH $\approx$ 0.8, and the period of the pH oscillation was approximately 67 s (Fig. 1). Moreover, the peak-to-peak ΔpH based on $\Delta V_{{{\text{out}}}}^{{{\text{peak}}}}$ and the oscillation period depended on the concentration of the BZ solution, as shown in Fig. 2. The initial concentration ratio for each component in the BZ reaction was constant, regardless of the concentration of the BZ solution (1/2 $\times$ *χ*, *χ*, and 2 $\times$ *χ*). At the lower concentration (1/2 $\times$ *χ*), the pH oscillation was observed upon adding malonic acid, but it was gradually attenuated over time owing to the consumption of the substrates (Fig. S2A). On the other hand, the pH oscillated more rapidly and lasted longer at the higher concentration (2 $\times$ *χ*). This is because there were sufficient amounts of reactants in the BZ solution. Thus, a linear relationship between the peak-to-peak ΔpH and the period of pH oscillation was found, depending on the concentration of the BZ solution. That is, the pH oscillation induced by the BZ reaction was clearly demonstrated even under the strongly acidic condition because the ISFET sensor can selectively detect the change in pH. However, we should investigate further to more convincingly show how the pH oscillation is involved in other reactions such as a cyclic redox reaction involving metallic catalysts.

The redox reaction of the ruthenium ion complex on the Pt electrode is

3 $${\text{Ru}}\left( {{\text{bpy}}} \right)_{3}^{3 + } + {\text{ e}}^{ - } {\text{Ru}}\left( {{\text{bpy}}} \right)_{3}^{2 + } .$$

From the Nernst equation, the electrode potential $E$, which is induced between the Pt electrode and the reference electrode, is expressed by

4 $$E = E_{0} + 2.303\frac{RT}{F}{\text{log}}\frac{{\left[ {{\text{Ru}}\left( {{\text{bpy}}} \right)_{3}^{3 + } } \right]}}{{\left[ {{\text{Ru}}\left( {{\text{bpy}}} \right)_{3}^{2 + } } \right]}}$$

where $E_{0}$ is the standard electrode potential, $R$ is the gas constant, $T$ is the absolute temperature, and $F$ is the Faraday constant. Therefore, the potential based on the BZ reaction is shown by

5 $$E - E_{0} \propto {\text{log}}\frac{{\left[ {{\text{Ru}}\left( {{\text{bpy}}} \right)_{3}^{3 + } } \right]}}{{\left[ {{\text{Ru}}\left( {{\text{bpy}}} \right)_{3}^{2 + } } \right]}} .$$

**Figure 3:**
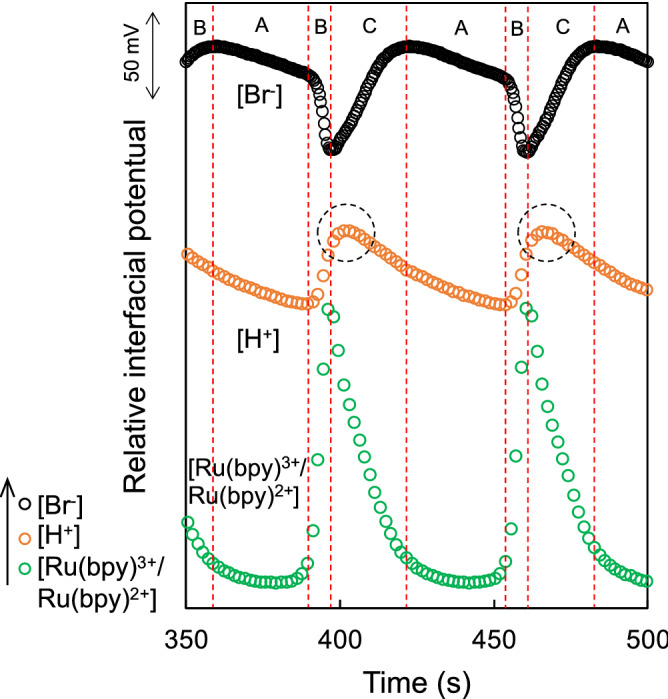
Self-oscillation behaviors of [H^+^], [Ru(bpy)^3+^/Ru(bpy)^2+^], and [Br^-^] based on relative interfacial potential. The durations of Processes A, B, and C based on the FKN mechanism (Table S2) are delineated by the dotted lines (red). The dotted circle (black) shows the position of the peak [H^+^].

Indeed, the redox potential of the ruthenium ion complex oscillated with a period consistent with that of [H^+^] and [Br^−^] oscillations, as shown in Fig. 3. [Br^-^] was simultaneously measured using a bromide-selective electrode (8005-10C, HORIBA). The redox reaction of the ruthenium ion complex was also confirmed by the change in the color of the BZ solution (Fig. S3). That is, the G component in the RGB color analysis was oscillated, similarly to the redox potential oscillation (orange ${\text{Ru}}\left( {{\text{bpy}}} \right)_{3}^{2 + }$ from/to light-green ${\text{Ru}}\left( {{\text{bpy}}} \right)_{3}^{3 + }$). Considering the Field, Koros, and Noyes (FKN) mechanism^9^, the BZ reaction takes place via three processes (A, B, and C), as shown in Table S2. The inhibitor Br^–^ is removed in Process A; the activator species HBrO_2_ is produced autocatalytically and the catalyst is oxidized in Process B; and the inhibitor Br^–^ is reformed upon reduction of the catalyst in Process C. From Fig. 3, the timing of peak potentials involved in $[{\text{Ru}}\left( {{\text{bpy}}} \right)_{3}^{3 + /2 + } ]$ and [Br^–^] were in reasonable accordance with the three processes. On the other hand, the increase and decrease in [H^+^] did not appear to follow three processes, showing a slight deviation of peak potentials (i.e., the phase shift) from those involved in $[{\text{Ru}}\left( {{\text{bpy}}} \right)_{3}^{3 + /2 + } ]$ and [Br^–^] (dotted circle in Fig. 3), although each period was consistent. Considering each reaction in the three processes and the sequence (e.g., Processes A → B → C) shown in Table S2, the sequence for the change in [H^+^] also seemed to shift one by one (i.e., Processes B → C → A). That is, the pH oscillation could not be simply explained from the reaction equations shown for the FKN mechanism. In the pH oscillation obtained in the FET measurements, malonic acid was added to the BZ presolution (pH 1.0). Then, dissociated malonic acids may have resulted in the change in [H^+^] in the BZ solution with lower pH, showing its equilibrium reaction $\left[ {{\text{CH}}_{2} \left( {{\text{COOH}}} \right)_{2} \rightleftarrows {\text{CH}}_{2} \left( {{\text{COOH}}} \right)\left( {{\text{COO}}^{ - } } \right) + {\text{H}}^{ + } \rightleftarrows {\text{CH}}_{2} \left( {{\text{COO}}^{ - } } \right)_{2} + 2{\text{H}}^{ + } ,{\text{ pK}}_{{\text{a}}} { }2.83{\text{ and }}5.69} \right]$. In fact, Δ*V*_out_ ([H^+^]) gradually decreased after the addition of malonic acid, which resulted in pHs 2–3 with the generation of ${\text{BrCH}}\left( {{\text{COOH}}} \right)_{2}$ before Δ*V*_out_ ([H^+^]) first increased (the pH oscillation was observed) (Figs. 1 and S2). Therefore, the equilibrium reaction of malonic acid may be considered for the pH oscillation induced by the BZ reaction, on the basis of our consideration. However, the driving force for the increase in [H^+^] cannot be explained from this point. Basically, ruthenium ion complexes showed trivalent ${\text{Ru}}\left( {{\text{bpy}}} \right)_{3}^{3 + }$ (oxidation state) in the BZ presolution (pH 1.0), which was confirmed from its color (light-green), as shown in Fig. S1. That is, the increase in [H^+^] appeared to be related to the oxidation state of ruthenium ion complexes. In this case, the ruthenium ion complex–bromomalonic acid subsystem, the reaction steps of which are given below (reaction Eqs. 6 and 7), may be required for the increase in [H^+^] during the pH oscillation, considering the results of simulation reported in a previous paper^8^. Note that the oxidation reactions of ruthenium ion complexes, that is, the generation of ${\text{Ru}}\left( {{\text{bpy}}} \right)_{3}^{3 + }$, should have simultaneously proceeded in Process B at this time in this study.

6 $${\text{Ru}}\left( {{\text{bpy}}} \right)_{3}^{3 + } + {\text{BrCH}}\left( {{\text{COOH}}} \right)_{2} \rightleftarrows \left[ {{\text{Ru}}\left( {{\text{bpy}}} \right)_{3} \left( {{\text{III}}} \right){\text{BrCH}}\left( {{\text{COOH}}} \right)_{2} } \right]^{ + } + 2{\text{H}}^{ + }$$

7 $${\text{Ru}}\left( {{\text{bpy}}} \right)_{3}^{3 + } + \left[ {{\text{Ru}}\left( {{\text{bpy}}} \right)_{3} \left( {{\text{III}}} \right){\text{BrCH}}\left( {{\text{COOH}}} \right)_{2} } \right]^{ + } \rightleftarrows {\text{tartraric acid}} + 2{\text{Ru}}\left( {{\text{bpy}}} \right)_{3}^{2 + } + {\text{Br}}^{ - } + {\text{H}}^{ + }$$

This assumption also provides the generation of ${\text{Ru}}\left( {{\text{bpy}}} \right)_{3}^{2 + }$ as well as H^+^, which may explain the slight deviation of peak potentials in [H^+^] (dotted circle in Fig. 3). From the above considerations, malonic acid with higher pH was added to the BZ presolution with lower pH and then the dissociated malonic acid underwent H^+^ on the basis of its equilibrium reaction, resulting in the decrease in [H^+^] (Process X). Subsequently, undissociated malonic acid reacted with ${\text{Br}}_{2}$, which generate ${\text{BrCH}}\left( {{\text{COOH}}} \right)_{2}$, in accordance with the usual process. ${\text{BrCH}}\left( {{\text{COOH}}} \right)_{2}$ was incorporated in the ruthenium ion complex–bromomalonic acid subsystem, resulting in [H^+^] increase (Process Y). After the increase in [H^+^] based on this assumption, malonic acid spontaneously maintained its equilibrium reaction to reduce [H^+^] again (Process X). This is why Processes X and Y may have periodically occurred in turn; that is, the pH oscillation may have been electrically observed using the ISFET sensor. However, the peak pH and the period of pH oscillation simulated in a previous work gradually increased over time^8^, whereas in this study they were almost constant (Figs. 1 and S2). Therefore, such a pH oscillation induced by the BZ reaction should be further simulated in the future to elucidate the reaction mechanism. Although there have already been numerous studies in which the reaction rates of catalysts and intermediates were analyzed, changes in [H^+^] were not considered.

### pH oscillation imaging with arrayed-gate ISFET sensor

The large-scale and high-density ISFET sensor (arrayed-gate ISFET sensor) chip, which was developed on the basis of CMOS integrated circuit technologies^34^, enabled the two-dimensional imaging of pH oscillation induced by the BZ reaction. Figure S4 shows Δ*V*_out_ with the change in pH from 4.01 to 9.18, detected using the arrayed-gate ISFET sensor. The data shown in Fig. S4A were obtained from three ISFETs, which were randomly chosen from among the 256 $\times$ 256 ISFETs. Δ*V*_out_ changed immediately after changing the pH buffer, indicating the good responsivity to the change in pH. Δ*V*_out_ shifted in the negative direction when pH was changed from 1.68 to 10.01, and then Δ*V*_out_ returned to the original value in a stepwise manner for the repeated pH measurements, similarly to the single-gate ISFET sensor. This result demonstrates that the Ta_2_O_5_ membrane as the gate insulator of the arrayed-gate ISFET sensor developed in this study remained electrochemically stable when immersed in solutions of various pHs and that each ISFET operated stably. Considering the data shown in Fig. S4A, a linear plot of Δ*V*_out_ vs pH was obtained from the 256 $\times$ 256 arrayed ISFETs, 0.5% of which were excluded from the evaluation owing to electrical failures, as shown in Fig. S4B. This calibration curve indicated a pH sensitivity of 55.6 mV/pH near the Nernstian response. Thus, the electrical responsivity of the arrayed-gate ISFET sensor to changes in pH was in good agreement with that of the single-gate ISFET sensor shown in Fig. S1. In addition, the arrayed-gate ISFET sensor measures two-dimensionally the change in pH, which is in contrast to the single-gate ISFET that measures the change in pH at a local point, and can be used as a pH image sensor.

**Figure 4:**
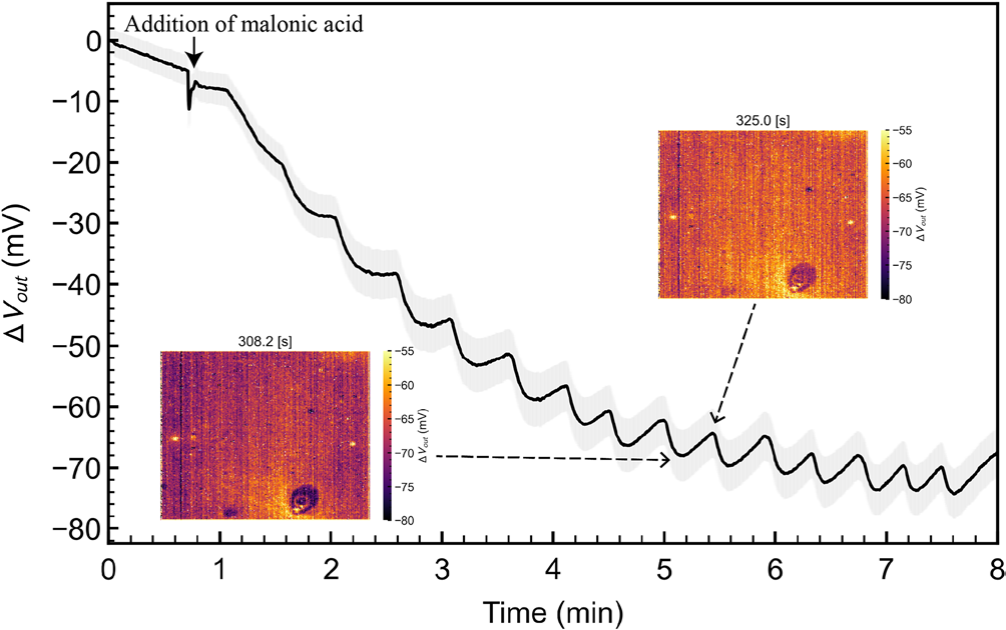
pH oscillation behavior on arrayed-gate ISFET sensor. Δ*V*_out_ was output for pH analysis. The grey band around the line graph shows the standard errors for the 256 $\times$ 256 ISFETs except for 0.5% electrical failures.

Using the arrayed-gate ISFET sensor, we monitored the pH oscillation induced by the BZ reaction in real time, as shown in Figs. 4 and S5. From Δ*V*_out_ for the arrayed-gate ISFET sensor, the pH (−log[H^+^]) oscillation was clearly observed upon adding malonic acid to the BZ presolution at the concentration of *χ* (Fig. 4), similarly to Δ*V*_out_ monitored by the single-gate ISFET (Fig. 1). In addition, the arrayed-gate ISFET sensor, as the pH image sensor, visually demonstrated the pH oscillation induced by the BZ reaction in real time via the change in the color, which corresponded to Δ*V*_out_, as shown in the movie (Fig. S5). The oscillation period was approximately 30–40 s, whereas $\Delta V_{{{\text{out}}}}^{{{\text{peak}}}}$ for the arrayed-gate ISFET sensor was approximately 10 mV (ΔpH $\approx$ 0.2) smaller than that for the single-gate ISFET, despite the similar trend of pH sensitivity. This may be due to the fact that the volume of the measurement solution in the arrayed-gate ISFET sensor was 1 mL smaller than that in the single-gate ISFET (10 mL); moreover, the effect of stirring of the BZ solution was different. Optimal agitation would be necessary to observe the periodic pH oscillation using the ISFET sensors. Thus, the chemical self-oscillation was imaged in real time on the basis of the change in pH using the CMOS integrated circuit device. In addition, to observe the patterns similar to a series of expanding concentric rings of the BZ reaction observed in petri dishes (target pattern)^39^, several arrayed-gate ISFET sensor chips may be arranged on a wider area in the future.

### Chemoelectrical self-oscillation of polymer brush with single-gate ISFET sensor

**Figure 5:**
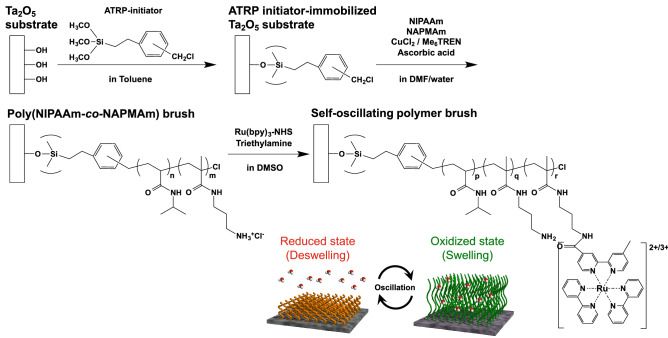
Poly(NIPAAm-*r*-NAPMAm-*r*-Ru(bpy)_3_ NAPMAm) brush grafted on Ta_2_O_5_ gate surface by SI-ARGET ATRP. These illustrations were drawn using the software (ChemDraw ver. 13.0 and Shade 3D ver. 15).

**Figure 6:**
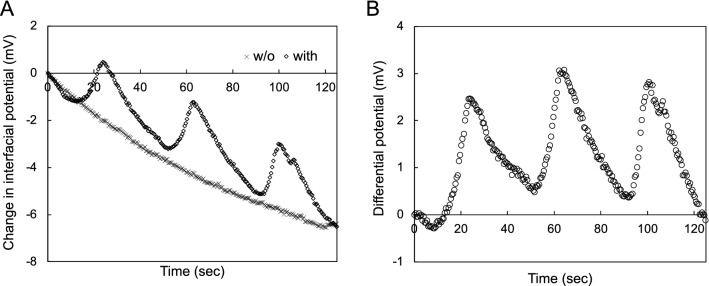
Electrical monitoring of self-oscillating polymer brush grafted on Ta_2_O_5_ gate insulator using single-gate ISFET sensor. (**A**) Electrical signals for single-gate ISFET sensors with and without self-oscillating polymer brush. (**B**) Differential signal based on (**A**).

As shown in the above sections, the solution-gated ISFET sensor can directly detect the pH oscillation induced by the BZ reaction on the Ta_2_O_5_ gate insulator with hydroxy groups, which show the equilibrium reaction with hydrogen ions. On the other hand, such a sensing surface is chemically modified and functionalized to detect target samples by ion-sensitive membranes and biomolecular recognition sites such as DNA probes and enzymes^22,40^. The chemical modification on the Ta_2_O_5_ gate insulator contributes to the specific and selective detection of target ions and biomolecules at the expense of losing the original pH sensitivity owing to the blocking of hydroxy groups. In particular, the thermoresponsive poly(*N*-isopropylacrylamide) (PNIPAAm) brushes grafted on the Ta_2_O_5_ gate insulator were evaluated as a bioelectrical interface for biosensing on the basis of the electrical property of the ISFET sensor. In this case, the difference between the capacitances of the PNIPAAm brushes at the swelling state and the deswelling state was found in previous studies.^37,41^ Moreover, the PNIPAAm gel copolymerized with ruthenium complexes (one of the catalysts) shows the self-oscillating behavior on the basis of the BZ reaction with the redox reaction of complexes^5,18^. That is, the self-oscillating behavior based on the BZ reaction can also be observed as a change in the capacitance of the PNIPAAm brush with ${\text{Ru}}\left( {{\text{bpy}}} \right)_{3}^{3 + /2 + }$ grafted on the Ta_2_O_5_ gate insulator, using the ISFET sensor. The self-oscillating polymer [designed to be a random copolymer containing NIPAAm, Ru(bpy)_3_, and NAPMAm as binding sites of Ru(bpy)_3_], that is, the poly(NIPAAm-*r*-NAPMAm-*r*-Ru(bpy)_3_ NAPMAm) brush, was successfully grafted on the Ta_2_O_5_ gate insulator of the ISFET sensor by SI-ARGET ATRP, followed by the conjugation of Ru(bpy)_3_-NHS ester (Fig. 5). After cleaning the Ta_2_O_5_ gate surface, ATRP initiators were tethered at the surface with hydroxy groups by silane coupling. Then, NIPAAm and NAPMAm monomers were copolymerized by grafting from the surface with the initiators for 20 h to control the thickness of poly(NIPAAm-*r*-NAPMAm) in the aqueous solution with the mixture of reagents for ARGET ATRP. After the ARGET ATRP reaction, Ru(bpy)_3_-NHS ester was covalently reacted with amino groups of the poly(NIPAAm-*r*-NAPMAm) brush grafted on the Ta_2_O_5_ gate insulator. The thickness was measured to be approximately 20 nm in atmosphere by AFM (Fig. S6). Using the prepared device, we electrically monitored the self-oscillation of the polymer brush, as shown in Fig. 6. In the measurement, ruthenium ion complexes and malonic acid were not included in the BZ presolution at the concentration of *χ*, and then malonic acid only was added to the poly(NIPAAm-*r*-NAPMAm-*r*-Ru(bpy)_3_ NAPMAm) brush-grafted Ta_2_O_5_ gate surface. In particular, the glass substrate grafted with the same polymer brush was used to cover over the modified Ta_2_O_5_ gate surface to prevent the BZ components such as Br^-^ from diffusing away from the active surface, that is, to maintain the BZ reaction at the surface (Fig. S7). In fact, the poly(NIPAAm-*r*-NAPMAm-*r*-Ru(bpy)_3_ NAPMAm) brush-grafted Ta_2_O_5_ gate ISFET demonstrated electrical self-oscillation, as detected on the basis of the swelling–deswelling behavior of the polymer brush. On the other hand, the poly(NIPAAm-*r*-NAPMAm) without Ru(bpy)_3_ brush-grafted Ta_2_O_5_ gate ISFET (control sensor) showed no electrical responses, although the signals of both sensors gradually and slightly decreased owing to the change in pH upon the addition of malonic acid, as shown in Fig. 6A. Moreover, the difference between the signals of the two sensors (${\Delta }V_{{{\text{out}}}}^{{{\text{diff}}}}$) clearly showed the self-oscillation with an amplitude of approximately 3 mV and a period of 40 s (Fig. 6B). This self-oscillation signal was not caused by the change in pH, judging from the electrical behavior of the control sensor. Therefore, the electrical self-oscillation of the poly(NIPAAm-*r*-NAPMAm-*r*-Ru(bpy)_3_ NAPMAm) brush-grafted Ta_2_O_5_ gate ISFET should have resulted from the change in the capacitance of the polymer brush attributable to its swelling and deswelling behavior, judging from the results reported in previous papers^37,41^. From the view point of ionic charges, however, the modified ISFET sensor may have detected simultaneously the change in the charges of ruthenium ion complexes owing to the redox reaction (${\text{Ru}}\left( {{\text{bpy}}} \right)_{3}^{2 + } \rightleftarrows {\text{Ru}}\left( {{\text{bpy}}} \right)_{3}^{3 + }$) on the Ta_2_O_5_ gate surface, similarly to the principle of pH sensing (*see* “Conclusion” section). In this case, the effect of these charges on Δ*V*_out_ would have depended on the density of ruthenium ion complexes in the polymer brush grafted on the Ta_2_O_5_ gate surface. Also, the increase in the density of positive charges based on the oxidation reaction should have induced the increase in Δ*V*_out_, in accordance with the source–follower circuit employed in this study^41^. On the other hand, the effect of the change in the capacitance of the polymer brush on Δ*V*_out_ should be additionally considered for the self-oscillating polymer (i.e., swelling $\rightleftarrows$ deswelling). In previous works, the thermoresponsive PNIPAAm-brush-grafted Ta_2_O_5_ gate ISFET clearly showed Δ*V*_out_ for the change in the temperature, which induced the swelling–deswelling behavior of the PNIPAAm brush past the lower critical solution temperature (LCST), resulting in the change in the capacitance of the PNIPAAm brush^37,41^. Considering the discussion in previous works, the capacitance of the polymer brush in the swelling state would have been larger than that in the deswelling state owing to the hydration of the polymer brush, resulting in the increase in Δ*V*_out_. That is, Δ*V*_out_ of the poly(NIPAAm-*r*-NAPMAm-*r*-Ru(bpy)_3_ NAPMAm)-brush-grafted Ta_2_O_5_ gate ISFET should have increased on the basis of the phase transition caused by the oxidation of ${\text{Ru}}\left( {{\text{bpy}}} \right)_{3}^{2 + }$ to ${\text{Ru}}\left( {{\text{bpy}}} \right)_{3}^{3 + }$ (i.e., swelling). This indicates that the positive shift in Δ*V*_out_ for the self-oscillating polymer brush may have been synergistically induced by the increases in the density of ionic charges of ruthenium ion complexes (bivalent to trivalent ions) and the capacitance of the polymer brush with swelling, which were primarily induced by the oxidation reaction. Moreover, ruthenium ion complexes were covalently introduced into the polymer brush, which then enhanced the ionization of the polymer brush; therefore, the transition between hydration and dehydration appeared to smoothly occur at the poly(NIPAAm-*r*-NAPMAm-*r*-Ru(bpy)_3_ NAPMAm)-brush-grafted Ta_2_O_5_ gate surface (Fig. 6). The amount of output signals (i.e., the amplitude) and periods would have been mainly derived from the length and density of the polymer brush chain and the concentration of BZ solution. From the slope of ${\Delta }V_{{{\text{out}}}}^{{{\text{diff}}}}$ for its increase or decrease, the reaction rate for the oxidation appeared to be higher than that for reduction^5^. This indicates that the swelling behavior of the poly(NIPAAm-*r*-NAPMAm-*r*-Ru(bpy)_3_ NAPMAm) brush would have occurred more smoothly than its deswelling behavior at the Ta_2_O_5_ gate surface. Thus, the chemical self-oscillation behavior that resulted in the structural change of the polymer brush with the swelling–deswelling transition was successfully monitored as the electrical self-oscillating behavior using the ISFET sensor. This suggests that such chemical oscillations can generate electrical energies for future self-oscillating sensors and actuators.

## Conclusion

In this study, we demonstrated the electrical monitoring of self-oscillation behaviors at the chemoelectrical interface based on the BZ reaction between the electrolyte solution and the Ta_2_O_5_ gate insulator of the ISFET sensor. First, the pH oscillation induced by the BZ reaction was electrically monitored using the nonmodified single-gate and arrayed-gate ISFET sensors. This is because the ISFET sensors had the superior ability to detect ionic charges; otherwise, the oxide gate surface with hydroxy groups in the solution showed the equilibrium reaction with hydrogen ions with positive charges, depending on the pH at such a chemoelectrical interface. In particular, the arrayed-gate ISFET sensor provided images of the pH oscillation owing to the large-scale and high-density format. To clarify the mechanism of the BZ reaction with pH oscillation, the ruthenium ion complex-bromomalonic acid subsystem may be added to it, considering the simulation of the pH oscillation shown in a previous paper^8^. In addition, the equilibrium reaction of malonic acid should be considered to elucidate the BZ reaction mechanism including the pH oscillation owing to the relatively large difference between the pH of BZ presolution ($\approx 1)$ and the pKa of the malonic acid ($\approx 3)$ added. Second, the self-oscillating polymer brush, poly(NIPAAm-*r*-NAPMAm-*r*-Ru(bpy)_3_ NAPMAm), grafted on the Ta_2_O_5_ gate insulator by SI-ARGET ATRP, clearly exhibited the periodical electrical response of the ISFET sensor. This self-oscillation behavior was mainly based on the change in the capacitance of the polymer brush derived from its swelling–deswelling behavior based on the BZ reaction on the surface of Ta_2_O_5_ gate insulator, that is, at the chemoelectrical interface, although the effect of ionic charges may be considered as well.

## Methods

### Chemicals

The following chemicals and materials were used in this study. *N*-isopropylacrylamide (NIPAAm) was provided by KJ Chemicals (Tokyo, Japan) and purified by recrystallization in toluene/hexane. Tris[2-(*N*,*N*-dimetylamino)ethyl]amine (Me_6_TREN), *N*-3-(aminopropyl)methacrylamide (NAPMAm) hydrocholoride, Ru(bpy)_3_-*N*-hydroxysuccinimide (NHS) ester, methyl 2-chloropropionate (MCP), and l-ascorbic acid were purchased from Tokyo Chemical Industries (Tokyo, Japan). Nitric acid (HNO_3_), sodium bromate (NaBrO_3_), malonic acid [CH_2_(COOH)_2_], tris(bipyridine)ruthenium (II) chloride [Ru(bpy)_3_^2+^ 2Cl^-^], copper (I) chloride (CuCl), [(chloromethyl)phenylethyl]trimethoxysilane (CMPETMS), triethylamine, *N*,*N*-dimethylformamide (DMF), dimethyl sulfoxide (DMSO, super dehydrated), toluene, dehydrated toluene, acetone, and methanol were purchased from Wako Pure Chemical Industries (Osaka, Japan).

### Devices

We used two types of ISFET sensor for monitoring the pH oscillation induced by the BZ reaction in this study. One was the single-gate ISFET sensor, which was composed of a silicon-based n-channel depletion-mode FET with a Ta_2_O_5_/SiO_2_ (100 nm/50 nm) layer as the gate insulator with a width (*W*) of 340 μm and a length (*L*) of 10 μm, respectively (ISFETCOM Co., Ltd.). The other type was the arrayed-gate ISFET sensor, which was composed of 256 $\times$ 256 pixels with a Ta_2_O_5_ (300 nm) layer as the gate insulator. Also, the sensing area of one pixel was 1.42 $\times$ 1.42 mm^2^ and the pixel pitch was 2 $\times$ 2 mm^2^. Other sensor performance parameters are shown in Table S1 and described in a previous paper^34^.

The extended-gate ISFET sensor was used for monitoring a self-oscillation behavior of a polymer brush grafted to the Ta_2_O_5_ gate surface. In this case, Ta_2_O_5_ film (100 nm) with the surface area of 12 $\times$ 12 mm^2^ was sputtered on a Au (100 nm)/Cr (20 nm) electrode, which was connected to the gate of a silicon-based n-channel junction-type FET (K246-Y9A, Toshiba), as an extended-gate electrode, considering ease of surface treatments^42,43^.

The Ta_2_O_5_ thin film was used as the passivation layer to prevent the leakage of currents as well as the pH-responsive layer in an electrolyte solution. A polycarbonate ring of 10 or 20 mm inner diameter was encapsulated to pour measurement solutions onto the Ta_2_O_5_ gate surface (0.5 or 1 mL) as a measurement well. Also, the single-gate ISFET sensor was immersed in a 5 mL measurement solution in a beaker for monitoring the pH oscillation.

### Electrical measurement of pH oscillation induced by BZ reaction using ISFET sensors

To confirm the fundamental electrical properties of the single-gate ISFET sensor, its gate voltage (*V*_G_)–drain current (*I*_D_) electrical characteristics were measured using a semiconductor parameter analyzer (B1500A, Agilent). A change in *V*_G_ in *V*_G_–*I*_D_ electrical characteristics was estimated as a threshold voltage (*V*_T_) shift, which was evaluated at a constant *I*_D_ of 700 μA and a constant drain voltage (*V*_D_) of 2 V. A Ag/AgCl reference electrode with a KCl solution was connected to the measurement solution through a salt bridge. As the pH measurement solution, standard buffer solutions with pHs of 1.68, 4.01, 6.86, 9.18, and 10.01 (Wako Pure Chemical Industries, Ltd.) were prepared. The time course of the change in surface potential at the gate surface (Δ*V*_out_) was monitored using a source follower circuit^35^, with which the potential change at the interface between an aqueous solution and a gate insulator can be read out directly at a constant *I*_D_ (RadianceWare Inc.). In this study, *V*_D_ and *I*_D_ were set to 1 V and 700 μA, respectively. On the other hand, the pH responsivity of the arrayed-gate ISFET sensor was monitored from pH 1.68 to pH 10.01 at the frame speed of 3.4 fps in this study, in accordance with the measurement setup shown in a previous work^34^. The measurement data (Δ*V*_out_) was automatically transformed into output images using a measuring software installed on a PC. For both ISFET sensors, Δ*V*_out_ was calibrated on the basis of the change in pH to analyze the pH sensitivity.

The BZ solution was composed of 1 M HNO_3_, 1 M NaBrO_3_, 1 M CH_2_(COOH)_2_, and 1 mM Ru(bpy)_3_^2+^ 2Cl^-^ in deionized water, the concentration of which is expressed as *χ*. That is, the BZ solution with 1/2 $\times$ *χ* was adjusted to 0.5 M HNO_3_, 0.5 M NaBrO_3_, 0.5 M CH_2_(COOH)_2_, and 0.5 mM Ru(bpy)_3_^2+^ 2Cl^-^ for each component in deionized water, for example. In the electrical measurement, malonic acid was added to the BZ presolution without malonic acid and the BZ solutions were stirred under batch conditions. The total volume of the BZ solution was controlled to be 0.5, 1, or 5 mL, depending on the size of measurement wells. The pH oscillation induced by the BZ reaction was observed on the basis of Δ*V*_out_ of both ISFET sensors without chemical modifications.

### Surface modification of polymer brush on Ta_2_O_5_ gate surface

#### Chemical modification of atom transfer radical polymerization (ATRP) initiator on Ta_2_O_5_ gate surface.

The self-oscillation behavior of a polymer brush grafted to the Ta_2_O_5_ gate surface was examined using the extended-Ta_2_O_5_-gate ISFET sensor, similarly to the single-gate ISFET sensor. The ATRP initiator (CMPETMS) was chemically modified on the Ta_2_O_5_ gate surface by the silane coupling reaction. The Ta_2_O_5_ gate surface was cleaned by oxygen plasma irradiation, followed by washing with ethanol and deionized water. The cleaned Ta_2_O_5_ gate substrate was placed into a separable flask, in which the relative humidity was 60%, for 2 h. Toluene solution containing CMPETMS (1.2 v/v%) was poured into the separable flask, and the solution was stirred for 20 h at 25 °C. The Ta_2_O_5_ gate surface with the ATRP initiator was washed with toluene and acetone and dried in a vacuum oven for 2 h at 110 °C.

#### Grafting of poly(NIPAAm-*r*-NAPMAm) brushes on Ta_2_O_5_ gate surface with initiator by surface-initiated activators regenerated by electron transfer (SI-ARGET) ATRP.

The NIPAAm monomer (5.65 g, 50 mmol) and NAPMAm hydrochloride (0.895 g, 5 mmol) were dissolved in a mixed solvent of deionized water (20 mL) and DMF (20 mL). CuCl_2_ (13.5 mg, 0.135 mmol) and Me_6_TREN (267.5 μL, 1 mmol) were added to the solution. The solution was stirred for 15 min to obtain the CuCl_2_/Me_6_TREN catalyst system. The prepared ATRP solution was poured into a separable flask containing the ATRP-initiator-tethered Ta_2_O_5_ gate insulator. MCP (21.5 μL, 0.20 mmol), which acts as an unbound sacrificial ATRP initiator, was then added to the solution. Finally, l-ascorbic acid (176 mg, 1 mmol) was added to the solution, so that the ARGET ATRP reaction proceeded for 20 h at 25 °C in the flask sealed with parafilm. After the ARGET ATRP reaction, the poly(NIPAAm-*r*-NAPMAm) brush-grafted Ta_2_O_5_ gate surface was thoroughly washed by ultrasonication in methanol and deionized water.

#### Addition of Ru(bpy)_3_-NHS ester to poly(NIPAAm-r-NAPMAm) brushes

Ru(bpy)_3_-NHS ester (40.4 mg, 0.20 mmol) and triethylamine (16.8 mL, 0.121 mM) were dissolved in DMSO (200 mL). 50 mL of the mixed solution was poured onto the poly(NIPAAm-*r*-NAPMAm)-brush-coated Ta_2_O_5_ gate surface. The reaction of Ru(bpy)_3_-NHS esters with amines at NAPMAm was induced for 4 h at 25 °C, and then the poly(NIPAAm-*r*-NAPMAm-*r*-Ru(bpy)_3_ NAPMAm)-grafted Ta_2_O_5_ gate surface was thoroughly washed by ultrasonication in methanol and deionized water. The thickness was measured in atmosphere by atomic force microscopy (AFM).

## Supplementary Information

Supplementary Information 1.

Supplementary Video 1.
