# Supplementary material for: Self-oscillating chemoelectrical interface of solution-gated ion-sensitive field-effect transistor based on Belousov–Zhabotinsky reaction
Source: Sci Rep. 2022 Feb 22;12:2949. doi: 10.1038/s41598-022-06964-4 (PMC8863790; doi:10.1038/s41598-022-06964-4)
Supplement: Supplementary file 1 — Supplementary Information 1. [file 41598_2022_6964_MOESM1_ESM.docx]

**Supporting Information**

**Self-oscillating chemoelectrical interface of solution-gated ion-sensitive field-effect transistor based on Belousov–Zhabotinsky reaction**

Toshiya Sakata,^*1^ Shoichi Nishitani,^1^ Yusuke Yasuoka,^1^ Shogo Himori,^1^ Kenta Homma,^1^ Tsukuru Masuda,^2^ Aya Mizutani Akimoto,^1^ Kazuaki Sawada,^3^ and Ryo Yoshida^1^

^1^ Department of Materials Engineering, School of Engineering, The University of Tokyo, 7-3-1 Hongo, Bunkyo-ku, Tokyo 113-8656, Japan

^2^ Department of Bioengineering, School of Engineering, The University of Tokyo, 7-3-1 Hongo, Bunkyo-ku, Tokyo 113-8656, Japan

^3^ Department of Electrical and Electronic Information Engineering, Toyohashi University of Technology, Tempaku-cho, Toyohashi 441-8580, Japan

*Corresponding author. E-mail: [sakata@biofet.t.u-tokyo.ac.jp](mailto:sakata@biofet.t.u-tokyo.ac.jp)

CORRESPONDING AUTHOR FOOTNOTE: Affiliation; Department of Materials Engineering, School of Engineering, The University of Tokyo, 7-3-1 Hongo, Bunkyo-ku, Tokyo 113-8656, Japan, TEL; +81-3-5841-1842, FAX; +81-3-5841-1842

**Sensor performance parameters of arrayed-gate ISFET sensor**

**Table S1**. Summary of sensor performance parameters.

**Fundamental electrical properties of single-gate ISFET sensor**

**Figure S1.** (A) pH response of nonmodified single-gate ISFET sensor. From the calibration line (inset), the single-gate ISFET sensor used in this study was approximately 53 mV/pH. The change in the interfacial potential surrounded by the dotted line was also caused by the addition of each component for the BZ presolution without malonic acid, the pH of which was approximately 1. (B) Change in interfacial potential upon adding each component of BZ solution obtained from (A). At 10 min, the measurement solution was totally exchanged to HNO_3_ (1 M, 150 μL), subsequently NaCl (1 M, 75 μL) was added instead of NaBrO_3_ and then deionized water (125 μL) was added. After that, Ru(bpy)_3_^2+^ 2Cl^-^ (1 mM, 50 μL) was added [*see* the dotted circle and arrow (left)]. The color of the mixed solution was orange for Ru(bpy)_3_^2+^ added to it. On the other hand, when NaBrO_3_ was included in the BZ presolution, the mixed solution was light-green for Ru(bpy)_3_^3+^ owing to the oxidation reaction [*see* the dotted circle and arrow (right)]. The pH of the BZ presolution was almost 1.

**pH oscillation at 1/2 × χ and 2 × χ using single-gate ISFET sensor**

**Figure S2.** Change in interfacial potential (Δ*V*_out_) oscillation based on BZ reaction at 1/2 $\times$ *χ* (A) and 2 $\times$ *χ* (B). Δ*V*_out_ was regarded as the change in [H^+^] (pH), considering the pH responsivity of single-gate ISFET sensor used in this study.

**Change in color of BZ solution based on redox reaction of ruthenium bipyridine complex Ru(bpy)_3_^2+/3+^**

**Figure S3.** Change in color of BZ reaction at a concentration *χ*. The cyclic redox reaction was observed from the change in the color of the BZ solution [Ru(bpy)_3_^2+^ (orange) $\rightleftarrows$ Ru(bpy)_3_^3+^ (light green) + e^-^]. This analysis was based on Red-Green-Blue (RGB) color model. Corresponding to the RGB analysis, the redox reaction of the ruthenium ion complex on the Pt electrode was measured in accordance with equations 4 and 5 (*see* “3. Results and Discussion”).

**Field, Koros, and Noyes (FKN) mechanism for BZ reaction**

**Table S2**. FKN mechanism

Reaction number Reaction

Process A ${5Br}^{-}+BrO_{3}^{-}+{6H}^{+}\rightleftarrows{3Br}_{2}+{3H}_{2}O$

1. $\mathrm{Br}^{-}+HOBr+H^{+}\rightleftarrows\mathrm{Br}_{2}+H_{2}O$
2. $\mathrm{Br}^{-}+HBrO_{2}+H^{+}\rightleftarrows2HOBr$
3. $\mathrm{Br}^{-}+BrO_{3}^{-}+{2H}^{+}\rightleftarrows HOBr+HBrO_{2}$

Process B 4$\mathrm{Ru}\left( \mathrm{bpy} \right)_{3}^{2+}+BrO_{3}^{-}+5H^{+}\rightleftarrows4Ru\left( \mathrm{bpy} \right)_{3}^{3+}+HOBr+{2H}_{2}O$

1. $2HBrO_{2}\rightleftarrows HOBr+BrO_{3}^{-}+H^{+}$
2. $\mathrm{HBr}O_{2}+BrO_{3}^{-}+H^{+}\rightleftarrows2BrO_{2}^{\cdot}+H_{2}O$
3. $\mathrm{Br}O_{2}^{\cdot}+Ru\left( \mathrm{bpy} \right)_{3}^{2+}+H^{+}\rightleftarrows HBrO_{2}+Ru\left( \mathrm{bpy} \right)_{3}^{3+}$

Process C $10Ru\left( \mathrm{bpy} \right)_{3}^{3+}+CH_{2}\left( \mathrm{COOH} \right)_{2}+BrCH\left( \mathrm{COOH} \right)_{2}+4H_{2}O$

$$\rightleftarrows10Ru\left( \mathrm{bpy} \right)_{3}^{2+}+\mathrm{Br}^{-}+2HCOOH+4CO_{2}+11H^{+}$$

1. $\mathrm{Br}_{2}+CH_{2}\left( \mathrm{COOH} \right)_{2}\rightleftarrows BrCH\left( \mathrm{COOH} \right)_{2}+\mathrm{Br}^{-}+H^{+}$
2. $6Ru\left( \mathrm{bpy} \right)_{3}^{3+}+CH_{2}\left( \mathrm{COOH} \right)_{2}+2H_{2}O\rightleftarrows6Ru\left( \mathrm{bpy} \right)_{3}^{2+}+HCOOH+2CO_{2}+ 6H^{+}$
3. $4Ru{(bpy)}_{3}^{3+}+BrCH{(COOH)}_{2}+2H_{2}O\rightleftarrows4Ru{(bpy)}_{3}^{2+}+HCOOH+\mathrm{Br}^{-}+ 2CO_{2}+5H^{+}$

**Fundamental electrical properties of arrayed-gate ISFET sensor**

**Figure S4.** pH responsivity of arrayed-gate ISFET sensor. (A) Real-time monitoring of Δ*V*_out_ with changing in pH (pH 9.18🡪7.41🡪6.86🡪4.01). Three ISFETs were randomly chosen from among the 256 × 256 ISFETs [(x, y): (10, 10), (128, 128), and (250, 250) for the positions]. (B) Calibration curve of Δ*V*_out_ with changing in pH for arrayed-gate ISFET sensor (256 $\times$ 256). This was based on (A). The standard errors ($\pm0.02 mV)$ were evaluated for the 256 $\times$ 256 ISFETs except for 0.5% electrical failures.

**Visualized pH oscillation with arrayed-gate ISFET sensor (*see* another file for the movie in SI)**

**
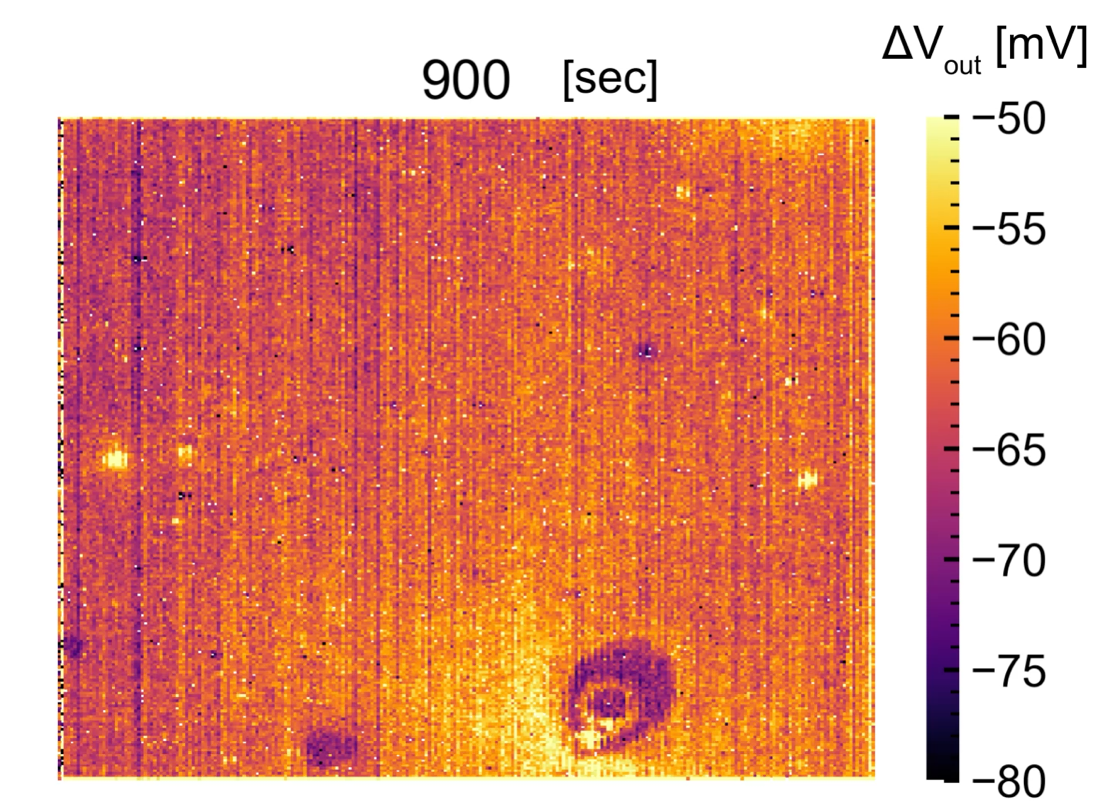
**

**Figure S5.** Movie of pH oscillation based on BZ reaction using arrayed-gate ISFET sensor with 256 $\times$ 256 pixels. Δ*V*_out_ corresponds to that shown in **Figure 4** [15–25 min (900–1500 s)]. Also, Δ*V*_out_ indicates the change in [H^+^] (pH). (*see* another file for the movie in SI; This movie was created using a software (Python ver. 3.9)

**Thickness of poly(NIPAAm-*r*-NAPMAm) brush grafted on the Ta_2_O_5_ gate insulator**

**Figure S6.** Thickness profile of poly(NIPAAm-r-NAPMAm) brush measured by AFM. The poly(NIPAAm-r-NAPMAm) brush was grafted on Ta_2_O_5_ surface by SI-ARGET ATRP. The thickness of the polymer film was analyzed to be approximately 20 nm against the unmodified Ta_2_O_5_ surface. The polymer film on the Ta_2_O_5_ surface was obtained by covering a part of the Ta_2_O_5_ surface with a polydimethylsiloxane (PDMS) sheet. The covered part was not modified in the subsequent modification processes, and the PDMS sheet was peeled off from the substrate after the polymerization.

**Measurement setup for self-oscillating polymer grafted on Ta_2_O_5_ gate surface**

**Figure S7.** Measurement setup for self-oscillating polymer grafted on Ta_2_O_5_ gate surface. The glass substrate grafted with the same polymer brush was used to cover over the modified Ta_2_O_5_ gate surface to prevent the BZ components such as Br^-^ from diffusing away from the active surface, that is, to maintain the BZ reaction at the surface. This illustration was drawn using a software (Microsoft PowerPoint 16.57).
